# Development of a New Amperometric Biosensor for Measurement of Plasma Galactose Levels

**DOI:** 10.1021/acsomega.3c06789

**Published:** 2024-02-07

**Authors:** Erhan Canbay, Ebru Sezer, Ebru Canda, Havva Yazıcı, Sema Kalkan Uçar, Mahmut Çoker, Eser Yildirim Sözmen

**Affiliations:** † Department of Medical Biochemistry, Faculty of Medicine, Ege University, Bornova, Izmir 35100, Turkey; ‡ Department of Pediatric Metabolic Disease, Faculty of Medicine, Ege University, Bornova, Izmir 35100, Turkiye

## Abstract

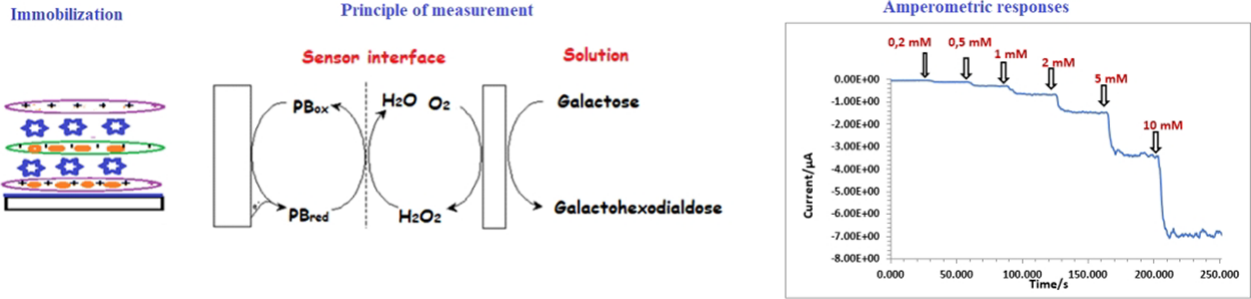

Galactosemia is an inherited disease that occurs as a result of insufficient or no synthesis of some enzymes (GALT, GALK, and GALE) in galactose metabolism. Failure to make an early diagnosis, especially in newborns, can lead to severe clinical and even fatal consequences. The aim of this study is to develop a biosensor for measuring free galactose in plasma. The immobilization components of the developed free galactose biosensor are screen printed carbon electrode (SCPE), Prussian blue (PB), chitosan (CHIT), Nafion (NAF), gold nanoparticle (GNP), and galactose oxidase (GaOX). The CHIT/GaOX/NAF-GNP/GaOX/CHIT-GNP/SCPE-PB electrode showed a sensitive amperometric response to detect galactose. While the surface characterization of the biosensor was performed with cyclic voltammetry and scanning electron microscopy, the optimization and performance characterizations were made by applying an amperometry technique. The amperometric operating potential for the free galactose biosensor was determined as −0.05 V. The linear detection range for the free galactose biosensor is between 0.025 and 10 mM. This range includes galactose levels in plasma of both healthy and patients. The percent coefficient of variation values calculated for intraday and interday repeatability of the developed biosensor are below 10%. The practical use of the biosensor, for which optimization and characterization studies were carried out, was tested in 10 healthy 11 patients with galactosemia, and the results were compared with the colorimetric method. In conclusion, the unique analytical properties and effortless preparation of the new galactose biosensor developed in this study make them serious candidates for point-of-care diagnostic testing.

## 1 Introduction

Galactosemia is a life-threatening autosomal recessive disease with a global frequency of one in every 62,000 live births.^1^ First described by Von Reuss in the early 1900s.^102^ Louis Leloir, who discovered the catabolism of galactose, won the Nobel Prize in Chemistry in 1970 for his discovery.^2,3^ For this reason, galactose catabolism is also known as the Leloir pathway. Three enzymes catabolize galactose in the Leloir pathway: galactokinase (GALK), galactose-1-phosphate uridyltransferase (GALT), and uridine diphosphate (UDP)-galactose 4-epimerase (GALE). In case of deficiency of one of the mentioned enzymes, galactose accumulates, and as a result galactosemia develops.

To improve life quality, continuous, fast, and precise monitoring systems are crucial for disease control, food safety, and environmental quality. Biosensors are promising devices for this purpose. Electrochemical sensors and biosensors are used in various fields, such as health, environmental monitoring, and food analysis, due to their high sensitivity, rapid response times, and cost-effectiveness.^4−8^ Enzymatic biosensors rely on enzymes as sensitive components for biomolecular recognition.^9^ Amperometry is a common technique that measures changes in current under a fixed potential, which is directly proportional to analyte concentration.^10,11^ Enzymatic biosensors typically exhibit excellent selectivity in practical applications due to the natural specificity of enzymes.

The layer by layer (LBL) technique, known as the layer-by-layer immobilization technique, is a very simple and practical method for the preparation of stable ultrathin multicomposite films by sequential adsorption of polyanions and polycations on the electrode surface.^12^ Developed by Decher, this technique was first developed for innate polyelectrolytes and later applied to structures such as proteins and enzymes.^13^ The film grows as the electrode interacts with positively and negatively charged polyelectrolytes and propagates through electrostatic interactions.^12^ Besides the possibility to control the amount of enzyme deposited at each step, the layer-by-layer method has an advantage over hydrogel entrapping in which the active ingredients are randomly dispersed.^12^ There are many biosensor studies in the literature using the LBL technique.^14,15^

Galactose oxidase (GaOx) contains a copper covalently bonded to water in its active center, two of which are tyrosine, two are histidine, and one is a solvent molecule.^16^ It catalyzes the stereospecific oxidation of d-isomers of a number of primary alcohol substrates such as GaOx, D-galactose, dihydroxyacetone (DHA), and polysaccharides with D-galactose at the reducing ends.^16^ Biosensors, in which enzymes are used as biocomponents, come to the forefront with their selectivity. For this purpose, GaOx enzyme is used in most of the galactose biosensors.^17^ The reaction catalyzed by GaOx is shown in eq 1:^17^
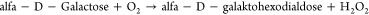
1

The high oxidation potential of H_2_O_2_ exposes the biosensor to the interference of electroactive compounds, such as uric acid and ascorbic acid. For this reason, redox mediators and enzymes, such as catalase and horseradish peroxidase (HRP) are frequently used in oxidase-based biosensors, which allow the detection of H_2_O_2_ at low potentials and reduce the interference effect.^18−22^ Electrochemical inorganic mediators that catalyze the oxidation or reduction of hydrogen peroxide^23,24^ have been preferred to HRP in the last 30 years. This causes the applied potential to decrease and, as a result, many electrochemical interferences to be avoided. In this perspective, hexacyanoferrates and especially Prussian blue (PB) (ferric hexacyanoferrate—PB) have found wide use. Also known as artificial peroxidase, PB has a high electrocatalytic activity against H_2_O_2_ and allows the determination of H_2_O_2_ at low potential.^25^

As is well-known and reported in the literature, the inherent micrometric porous structure of CHIT can increase the surface area and corresponding loading capacity of the sensors, thus contributing to amplification of the signal.^26^ In addition, when detecting relatively small molecules, the CHIT porous structures have minimal effect on analyte diffusion toward the electrodic surface.^27^ Finally, CHIT is nontoxic and environmentally friendly, a biodegradable and renewable raw material. Chitosan, which is a polycationic polymer, is also frequently used in LBL productions with this feature. In this study, the other component used in LBL formation is Nafion, a polyanionic polymer. Nafion, a perfluorosulfonic acid polymer, is often used as a semipermeable membrane to fabricate biosensors due to its excellent conductivity.^10,28,29^ Nafion (NAF) also increases the stability of biosensors with its biocompatible, adhesive properties.^30,31^ Nanostructured materials as immobilization elements are frequently used in biosensor construction with their high and active surface areas, mechanical and catalytic properties, as well as stability.^32^ Gold nanoparticles (GNPs), one of the most studied nanostructured materials, have received great attention due to their unique electronic, optical, and catalytic properties.^32^ GNPs, with their high conductivity, high surface/volume ratio, and good stability, stabilize the bioreceptors on the electrode surface and increase the efficiency and sensitivity of the biosensor without changing its structure.^33^

The aim of this study is to develop an inexpensive, easily prepared, tolerant of interference, and long-lasting galactose biosensor that can measure galactose levels in serum or plasma. The use of PB in biosensor fabrication allows for galactose measurement at lower potentials, while the electrostatic attraction forces between CHIT and Nafion form the main strategy for enzyme immobilization on the biosensor surface through a layer-by-layer method. GNP, on the other hand, is used to increase both the conductivity and surface area. Although there are galactose oxidase-based enzyme biosensors in the literature, a galactose biosensor utilizing CHIT, Nafion, and GNP in combination with a layer-by-layer immobilization method has not been encountered. The method used includes optimization ranging from the concentration of each component to the droplet volume, setting it apart from other studies in terms of this detailed work. Finally, the developed galactose biosensor was tested with real patient and healthy control plasma samples, which is another factor that distinguishes our study from other research.

## 2 Materials and Methods

### 2.1 Materials and Reagents

Galactose oxidase (source: Dactylium dendroide, ≥3000 units/g solid), chitosan (medium molecular weight), Nafion-117, potassium hexacyanoferrate (III), potassium hexacyanoferrate (II), potassium chloride, sodium chloride, hydrochloric acid, dipotassium hydrogen phosphate, monopotassium phosphate, galactose, galactose-1-phosphate, GNP solution (with a diameter of 5 nm), and other chemicals were obtained from Sigma-Aldrich (USA). In the experiments PalmSens Emstat3 potentiostat (Netherlands), screen printed carbon electrodes from Dropsens (DRP-C110, Switzerland) that contain carbon working electrode (4 mm diameter), carbon auxiliary electrode, and silver reference electrode were used.

A 0.1 M KCl containing 50 mM phosphate-buffered saline (PBS) buffer at pH 7.5 was used as a working buffer. To prepare the artificial serum, a mixture of 111 mM NaCl, 2.9 mM NaHCO_3_, 2.2 mM K_2_HPO_4_, 0.8 mM MgCl_2_, 2.5 mM urea, and 5 mM KCl was adjusted to pH 7.4.^34^ The galactose oxidase solution was prepared by dissolving it in 50 mM pH 7.5 PBS to a concentration of 80 mg/mL. The galactose solution was prepared by dissolving galactose in the artificial serum.

### 2.2 Sample Collection

The permission required for the collection of galactosemic and healthy control blood samples has been approved by the Ege University Faculty of Medicine Clinical Research Ethics Committee under decision number 20–7*T*/36. Plasma galactose levels necessary for the diagnosis of galactosemia are very high during the neonatal period but decrease with dietary intervention to levels lower but not as high as those of healthy individuals. Since capturing a galactosemic patient during the neonatal period is very challenging, plasma from healthy individuals were obtained by randomly adding standards to mimic the plasma galactose levels of both healthy and patient samples. Only one sample belonging to a galactosemic patient was obtained during the neonatal period. Blood samples collected in ethylenediaminetetraacetic acid tubes were centrifuged at 3000 rpm for 10 min. The supernatant (plasma) was transferred to an Eppendorf tube and stored at −40 °C until further analysis.

### 2.3 Fabrication of the Galactose Biosensor

**Figure 1:**
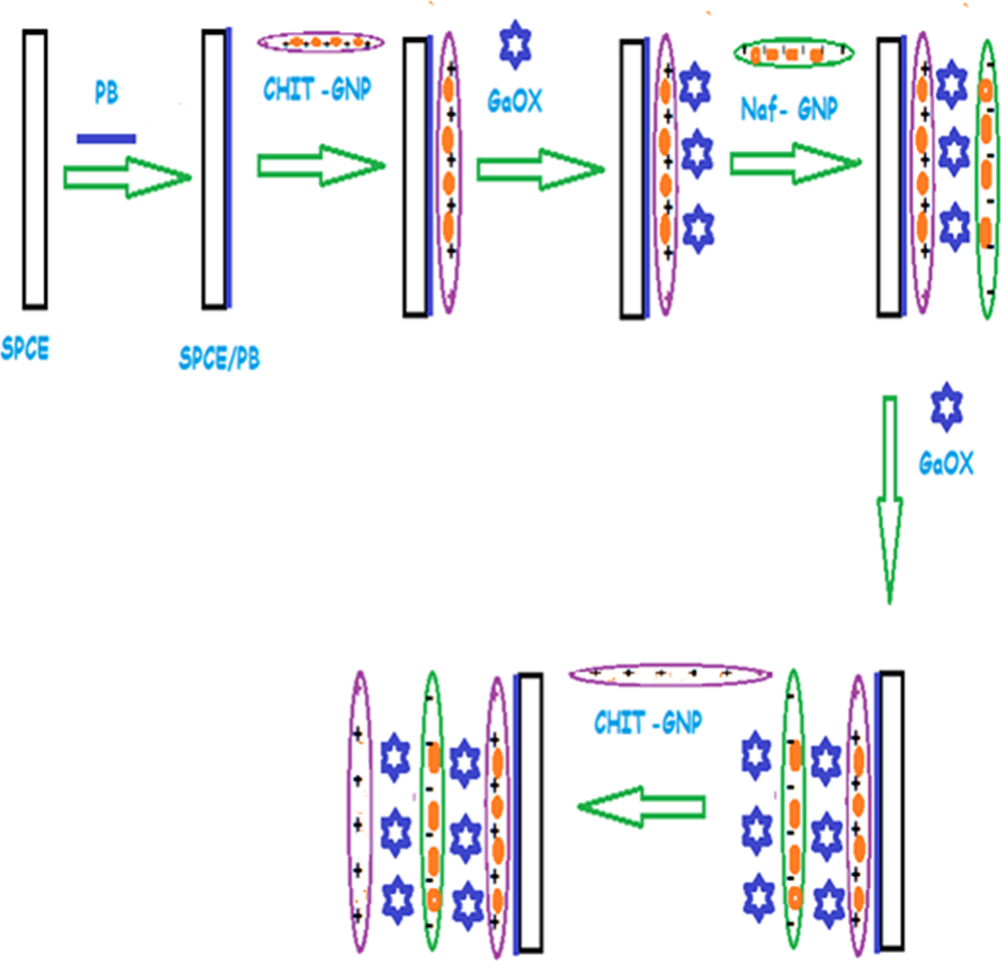
Preparation steps of the free galactose biosensor.

- In this study, free galactose biosensor was constructed by modifying the surface of screen-printed carbon electrode (SPCE). The schematic representation of the biosensor construction is given in Figure 1. The optimized biosensor preparation steps are as follows:
- SPCE/PB:^11^ SPCE is immersed in a saturated Na_2_CO_3_ solution and amperometrically scanned for 300 s at 1.20 V to remove contamination from the electrode surface. SPCE is immersed in a mixture containing 2.0 mM FeCl_3_, 2.0 mM K_3_Fe (CN)_6_, 0.1 M KCl, and 20 mM HCl. Twenty cycles are taken by scanning cyclic voltammetry between −0.2 and 1.0 V. It is then immersed in solution containing 0.1 M KCl and 20 mM HCl and scanned between −0.2 and 1.0 V until stable voltammograms are obtained. For stabilization, the PB-deposited SPCE is placed in an oven and dried at 100 °C for 30 min.
- SPCE/PB/CHIT-GNP: 5 μL of 0.1% chitosan solution, which has been prepared in 1% acetic acid, and GNP suspension are dropped onto the electrode surface. Then, the electrode is dried at room temperature.
- SPCE/PB/CHIT-GNP/GaOX: 5 μL of a 10 mg/mL suspension of galactose oxidase (GaOX) are dropped onto the electrode surface and allowed to dry at +4 °C.
- SPCE/PB/CHIT-GNP/GaOX/NAF-GNP: 5 μL of 0.1% Nafion solution is dropped on the SPCE/PB/CHIT-GNP/GaOX modified electrode surface and allowed to dry. 5 μL of GNP is added to the drying electrode, and the electrode is left to dry.
- SPCE/PB/CHIT-GNP/GaOX/NAF-GNP/GaOX: 5 μL of 10 mg/mL galactose oxidase (GaOX) suspension is dropped onto the electrode surface, and it is allowed to dry at +4 °C.
- SPCE/PB/CHIT-GNP/GaOX/NAF-GNP/GaOX/CHIT: 5 μL of CHIT solution is added to the modified electrode surface of SPCE/PB/CHIT-GNP/GaOX/NAF-GNP/GaOX, and it is allowed to dry at +4 °C. Finally, 50 μL of a 2.5% glutaraldehyde solution is added to the electrode surface and left for 20 min. After the incubation, the electrode surface is washed with distilled water.
  The fundamental mechanism in immobilization is the enzyme’s attachment to the electrode surface through electrostatic attraction between the cationic chitosan and the polyanionic matrix, namely, Nafion. The purpose of using GNP (graphene nanoparticle) here is twofold: to enhance conductivity and to provide a larger surface area for adsorbing more enzymes.

### 2.4 Principle of Measurement

**Figure 2:**
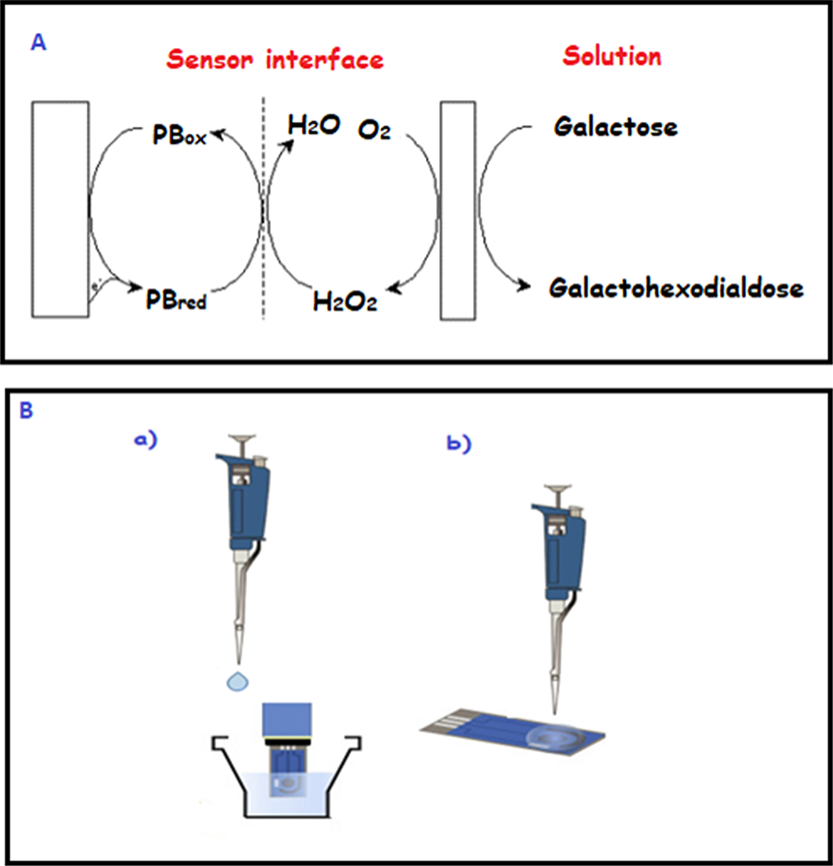
Working principle of the developed free galactose biosensor (A). (a) Stirred method and (b) drop-cast method (B).

The working principle of the developed free galactose biosensor is schematically shown in Figure 2. The oxidation of H_2_O_2_, an electroactive compound released as a result of galactose oxidase activity, constitutes the operating principle of many galactose oxidase biosensors. However, the oxidation potential of H_2_O_2_, an electroactive compound, is high, and it is susceptible to interference from various electroactive species, such as ascorbic acid and uric acid. Therefore, in this study, PB, a molecule capable of reducing H_2_O_2_ and enabling operation at lower potentials, was used on the electrode surface. Optimization studies revealed that the highest current differences for the response of the free galactose biosensor were achieved at −0.05 V. Therefore, optimization and characterization of the biosensor were conducted at this potential. The experiments were carried out amperometrically in pH 7.5, 50 mM PBS buffer containing 0.1 M KCl. Amperometric measurements were performed in two ways: stirred and drop-cast (Figure 2B). In the stirred method, 500 μL of galactose standards prepared in artificial serum were added to a measurement vessel containing 4.5 mL of buffer. During the measurement, the solution was stirred at 2000 rpm by using a magnetic stirrer (Figure 2B-a). In the drop-cast method, 25 μL of galactose standards prepared in artificial serum and 25 μL of buffer solution were mixed in an Eppendorf tube. From the final mixture, 50 μL of solution was dropped onto the electrode surface, and the measurement was taken at −0.05 V (Figure 2B-b).

### 2.5 Sample Analysis

A free galactose biosensor developed and a colorimetric galactose kit (Sigma, MAK012) were used to determine galactose levels in a total of 21 plasma samples, including 10 healthy and 11 galactosemic individuals (10 samples were from a pooled plasma pool, and 1 sample was obtained from an actual galactosemic patient). The results were compared. Additionally, plasma samples obtained from healthy individuals were spiked with standards to calculate the percent recovery for three levels: low (0.1 mM), medium (1 mM), and high (10 mM).

## 3 Results and Discussion

### 3.1 Findings on the Surface Morphology of the Modified Electrode

**Figure 3:**
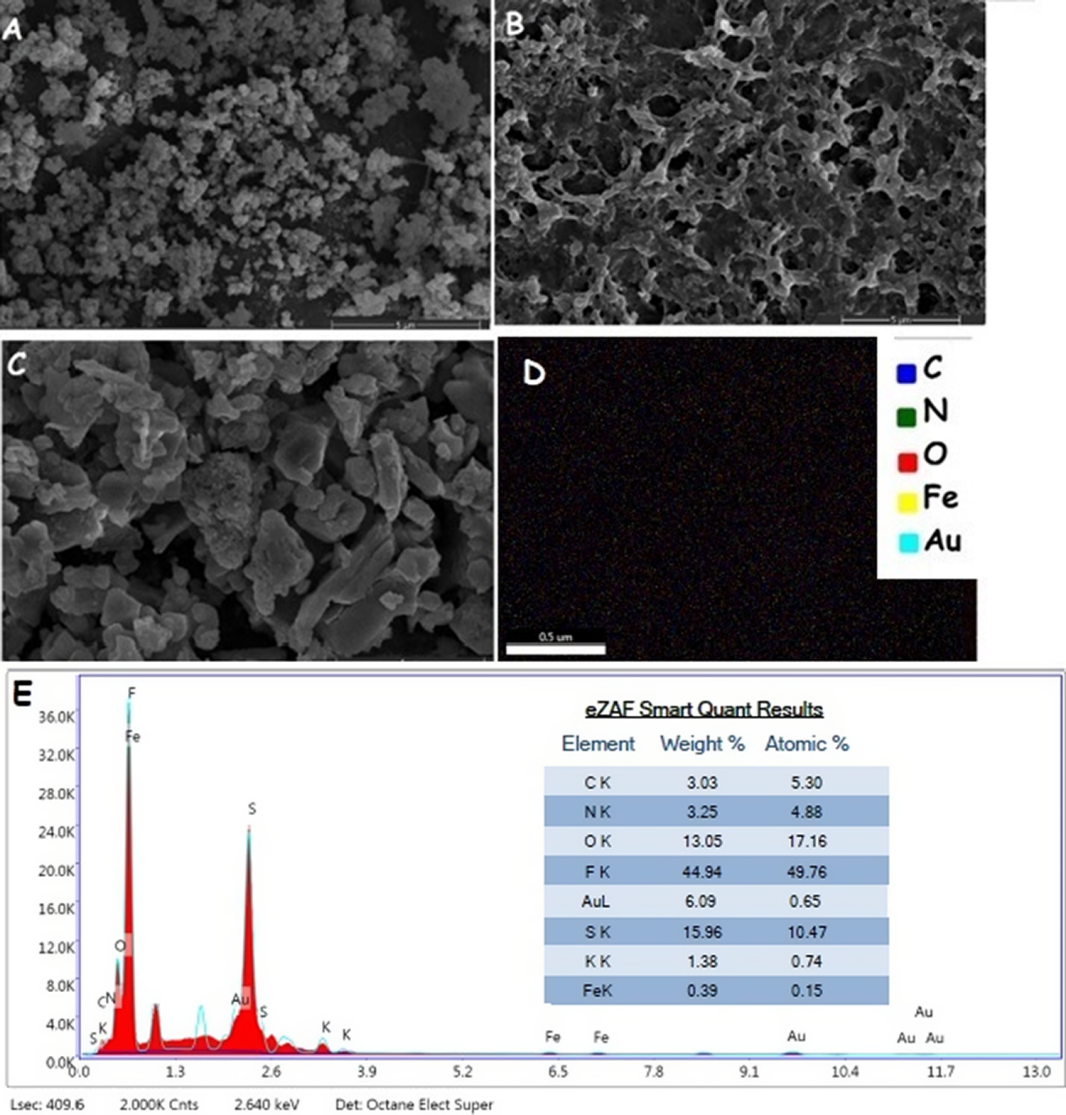
SEM images are of a SPCE/PB at 25,000× (A), a SPCE/PB/CHIT-GNP/NAF-GNP/CHIT at 25,000× (B), a SPCE/PB/CHIT-GNP/E/NAF-GNP/E/CHIT at 25,000× (C), EDS mapping, and spectrum of SPCE/PB/CHIT-GNP/NAF-GNP/CHIT (D and E).

Scanning electron microscopy (SEM) images of the modified electrodes can provide information about their surface morphologies. For this purpose, SEM images of SPCE/PB, SPCE/PB/CHIT-GNP/NAF-GNP/CHIT, and SPCE/PB/CHIT-GNP/GaOX/NAF-GNP/GaOX/CHIT electrodes were taken to visualize their surface structures, and the findings are shown in Figure 3. In Figure 3A, after PB electrodeposition, spherical PB particles are observed on the surface of the SPCE electrode. This image is in line with the literature and serves as additional evidence of PB accumulation on the electrode surface. Figure 3 represents the image of the enzyme-free electrode. The highly macroporous network structure, originating from chitosan and Nafion matrices, enhances mass transfer rate and provides a suitable area for enzyme immobilization. This porous structure (Figure 3C) becomes closed, and the layers thicken after enzyme immobilization, indicating the immobilization of the enzyme on the electrode surface. EDS and FE-SEM were used to confirm whether GNP, CHIT, and Nafion were successfully integrated on the electrode surface. Figure 3D, in particular, demonstrates a good distribution of C, O, N, Fe, and Au on the electrode surface. The spectrum in Figure 3E represents Au for GNP, Fe and K for PB, and C, N, and O for CHIT, while F and S represent Nafion. Therefore, the element spectrum indicates that the immobilization components were successfully integrated on the electrode surface.

### 3.2 Findings on the Electrochemical Behavior of Modified Electrodes

Cyclic voltammograms (CV) of the electrodeposition of PB are shown in Figure S1A. The obtained electrodeposition CVs are compatible with the literature (Nontipichet et al.^25^). In order to demonstrate the successful accumulation of PB on the electrode surface, measurements between-0.3 and 1.0 V were taken in 50 mM PBS using both a bare electrode (SPCE) and an electrode with accumulated PB (SPCE/PB). The results are shown in Figure S1B. Upon examining Figure S1B, it can be observed that there is no peak in the CV obtained with the bare electrode, while the characteristic peaks of PB are observed in the CV obtained with the SPCE/PB electrode. This indicates the successful accumulation of PB on the SPCE surface.

**Figure 4:**
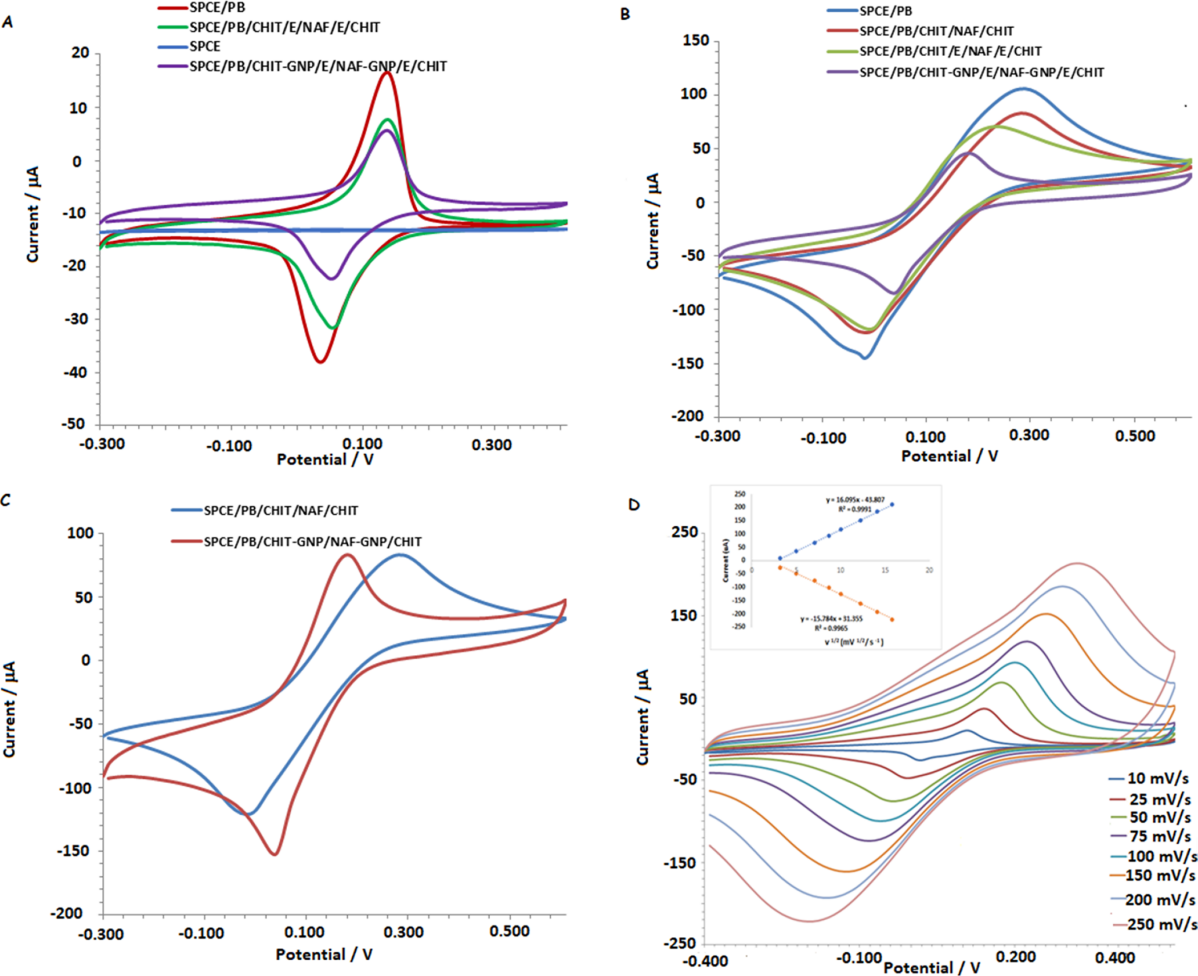
Cyclic voltammograms between −0.3 and 0.4 V taken at 50 mM pH:7.0 PBS with 0.1 M KCl with modified electrodes related to the immobilization stages (A). Cyclic voltammograms (50 mV/s) obtained in 5.0 mmol L^–1^ Fe(CN)_6_^3–/4–^ containing 0.1 M KCl were recorded for the immobilization stages (B). Cyclic voltammograms (50 mV/s) obtained in 5.0 mmol L^–1^ Fe(CN)_6_^3–/4–^ solution containing 0.1 M KCl were recorded for the purpose of determining the surface area provided by GNP (C). Cyclic voltammograms of the developed biosensor (SPCE/PB/CHIT-GNP/E/NAF-GNP/E/CHIT) were obtained at different scan rates in pH 7.5 PBS containing 0.1 M KCl and 5.0 mmol L^–1^ Fe(CN)_6_^3–/4–^ (D).

Figure 4A presents CVs obtained in 50 mM PBS between −0.3 and 0.4 V, illustrating the immobilization stages in biosensor fabrication. The immobilization of the enzyme onto the electrode surface resulted in a reduction in anodic and cathodic peak currents, which further decreased upon the addition of GNPs. This is because GNP enhances enzyme adsorption on the electrode surface through adsorption. The reduction can be attributed to the insulating nature of the enzyme and indicates its immobilization onto the electrode surface.

In Figure 4B, the findings related to the immobilization stages are shown through CVs obtained in a ferri/ferro solution. The addition of chitosan and Nafion to the electrode surface (red CV) lowered the peak currents of ferri/ferro due to the formation of a diffusion layer on the electrode surface. With the incorporation of the enzyme into this structure (green and purple CVs), the peak currents decreased even further. The reduction is more pronounced in the GNP-modified electrode, indicating more enzyme immobilization on the electrode surface.

To assess the impact of the surface area provided by GNP, CVs were obtained in ferri/ferro solution using enzyme-free SPCE/PB/CHIT/NAF/CHIT and SPCE/PB/CHIT-GNP/NAF-GNP/CHIT electrodes. The results are presented in Figure 4C. The electrode surface area was calculated using the Randles–Sevcik equation (Nontipichet et al.^25^; Wang et al.^45^):
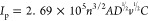
where *I*_p_,peak current; *n*, number of electrons transferred (*n* = 1); *D*, diffusion coefficient for ferricyanide (7.6 × 10^–6^ cm^2^/s); *v*, scan rate (V/s); and *C*, ferricyanide concentration (5.0 × 10^–6^ mol/cm^3^).

According to this equation, the surface area for the GNP-modified electrode was calculated as 0.183 cm^2^, while for the electrode without GNP, the surface area was calculated as 0.145 cm^2^. This finding demonstrates that GNP increased the surface area by 26%.

The electrochemical behavior of the SPCE/PB/CHIT-GNP/E/NAF-GNP/E/CHIT electrode was also investigated over a scanning rate range of 10–250 mV/s. Figure 4D displays the CVs of the modified electrode at different scanning rates. The redox peaks of PB increased as the scanning rate has increased. The linear relationship between the square root of the scanning rate and the anodic and cathodic peak currents indicates a diffusion-controlled process.

**Figure 5:**
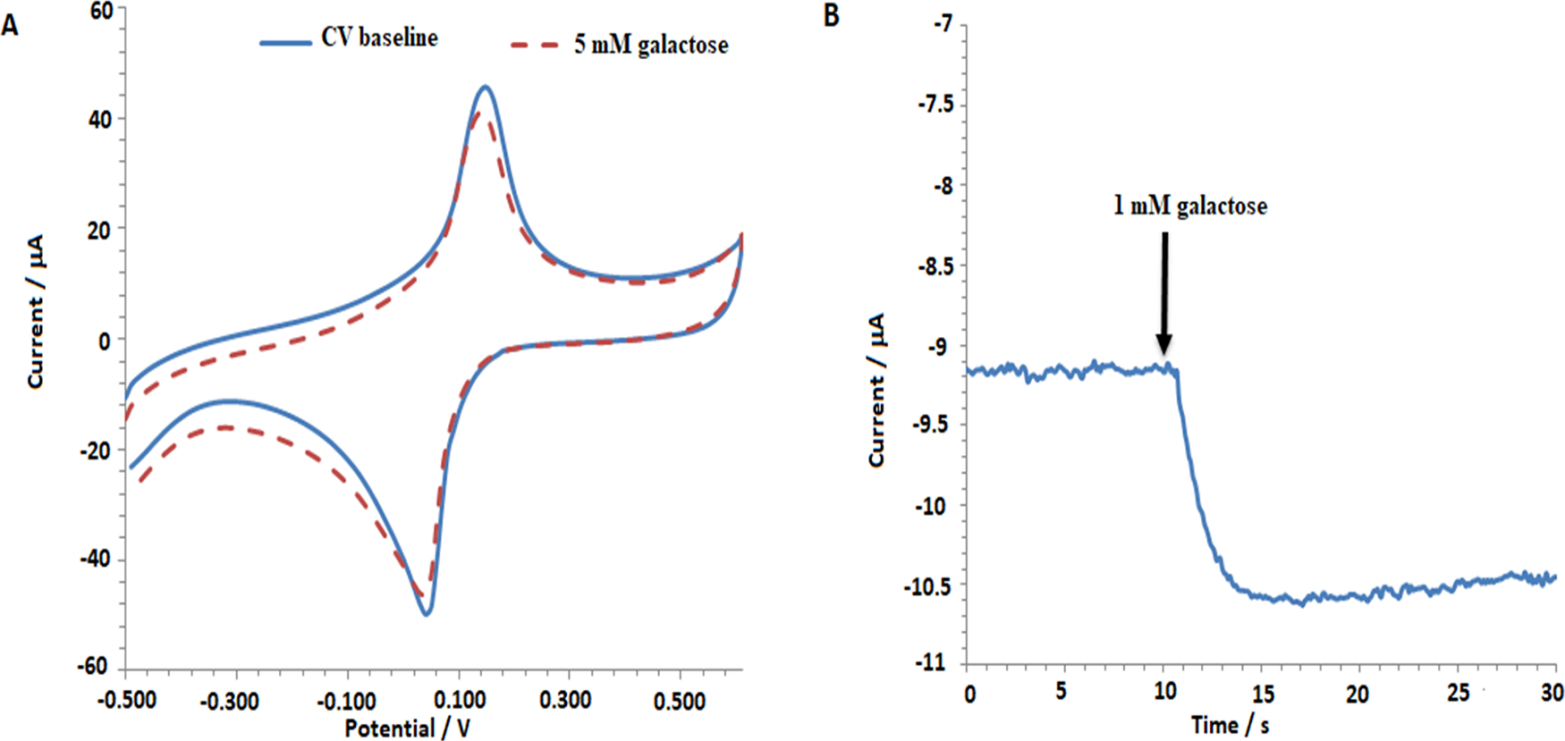
(A) Cyclic voltammograms of the galactose biosensor (SPCE/PB/CHIT-GNP/GaOX/NAF-GNP/GaOX/CHIT) in 0.1 M KCl containing 50 mM pH 7.5 PBS, with 0 and 5 mM galactose, and (B) amperometric results obtained at −0.05 V upon adding 1 mM galactose.

The findings regarding the galactose response of the developed and characterized biosensors are presented in Figure 5. Figure 5A displays the CV responses of the biosensor in the presence of 0 and 5 mM galactose, while Figure 5B shows amperometric responses obtained at −0.05 V upon the addition of 1 mM galactose. The working principle of the developed biosensor relies on the reduction of PB catalyzing the H_2_O_2_ generated by the enzymatic reaction on the electrode surface. Hence, differentiation in the cathodic peak of PB is expected, and this reduction in the cathodic peak is anticipated to result in a decrease in current upon galactose addition in the performed amperometric measurement. Indeed, in Figure 5, the cathodic peak decreased in the presence of galactose, and an instantaneous current difference created a response to galactose addition. The cathodic peak of PB lies between 0.2 and −0.2 V. To determine the amperometric working potential, amperometric measurements were taken at 0.05 V intervals. Based on this, the responses obtained at 0.0 and −0.05 V are very close to each other and relatively higher than the others. Therefore, the amperometric working potential was determined as −0.05 V. Optimization and characterization studies have been conducted through this potential and by sequential addition of 0.2–10 mM galactose concentration (Figure S2).

### 3.3 Optimization of the Bioactive Layer

#### 3.3.1 Findings on Determination of Immobilization Configuration

**Figure 6:**
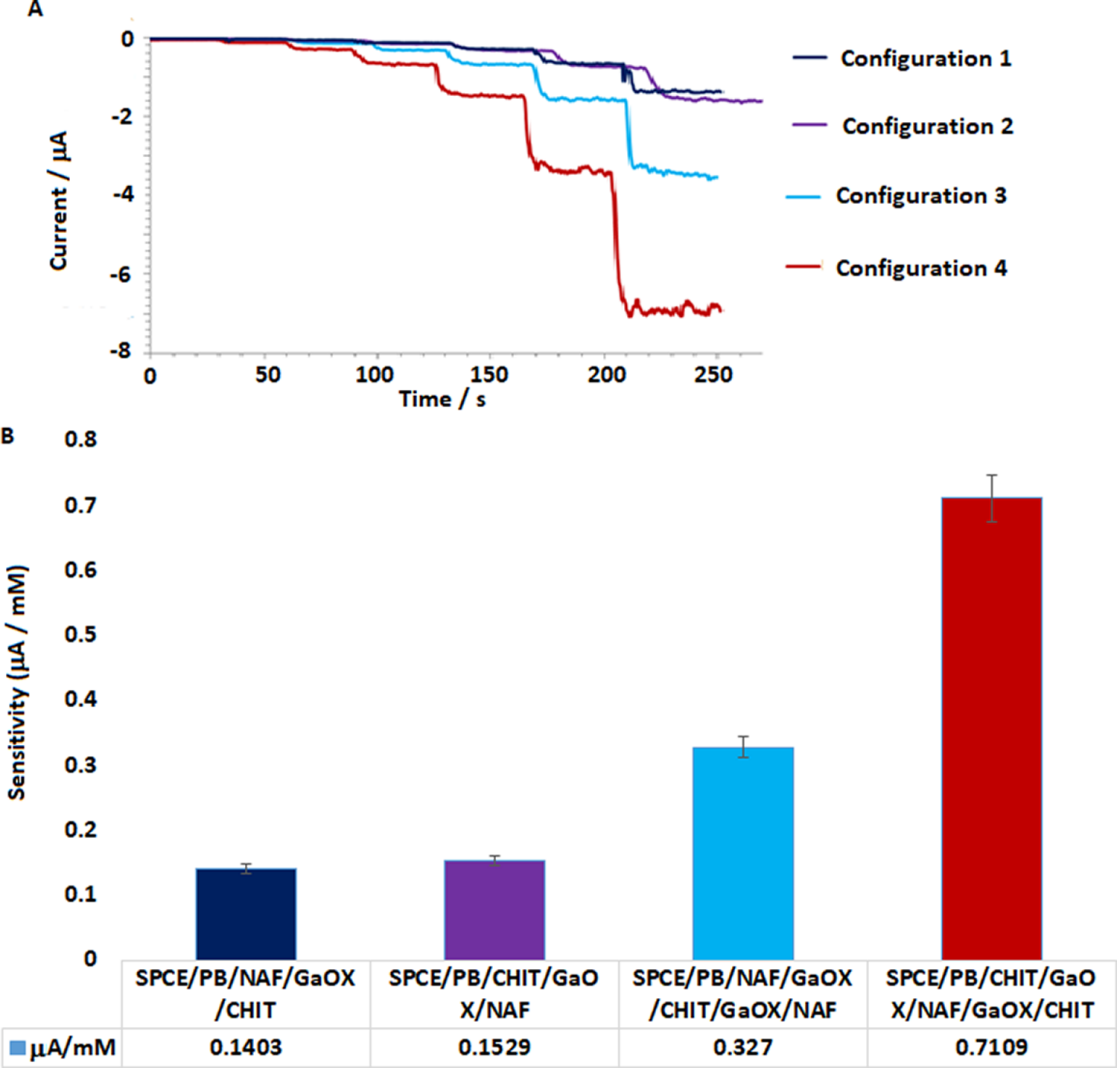
Graph shows amperometric responses (A) with different modified SPCEs for the determination of galactose concentration in the 0.2–10 mM concentration range and its corresponding bar graph (B).

In this study, the basis of the immobilization method is to immobilize the enzyme on the electrode surface through electrostatic forces between a polycationic component, chitosan, and a polyanionic component, Nafion. In essence, the aim is to immobilize the enzyme by using a layer-by-layer approach. Considering this, the first aspect to be investigated in the immobilization method is the electrode configuration. For this purpose, single-enzyme layer SPCE/PB/NAF/GaOX/CHIT, SPCE/PB/CHIT/GaOX/NAF, and double-enzyme layer SPCE/PB/NAF/GaOX/CHIT/GaOX/NAF, SPCE/PB/CHIT/GaOX/NAF/GaOX/CHIT electrodes were prepared and tested within the range of 0.2–10 mM galactose concentrations. The modified electrodes were compared in terms of sensitivity, and the results were plotted on a graph (Figure 6). Although a three-layer model was also tested, measurements could not be taken due to electrode structure disruption; therefore, they were not included in the comparison. The measurements were carried out through sequential additive amperometric measurements with five repetitions. The percent coefficient of variation (%CV) values for a concentration of 1 mM are as follows: 7.54; 15.21; 6.51; 2.36.

As clearly evident from Figure 6, the sensitivity of the two-layer electrodes is significantly higher compared with the single-layer ones. Among the two-layer models, the highest biosensor responses were obtained with the SPCE/PB/CHIT/GaOX/NAF/GaOX/CHIT modified electrode, where chitosan serves as the main immobilization component. Although the pore structure of chitosan is smaller than Nafion’s, the use of glutaraldehyde for cross-linking in the last layer of the electrodes where chitosan is the final layer enhances the repeatability of the biosensor. Lower biosensor responses and high variation coefficients observed in electrodes where Nafion is the final layer suggest that the enzyme might have been washed off the electrode surface during electrode rinsing. As a result, due to both higher sensitivity and stability in responses, the immobilization configuration SPCE/PB/CHIT/GaOX/NAF/GaOX/CHIT has been determined.

#### 3.3.2 Effect of Chitosan Amount on Biosensor Response

In this section of the study, while keeping other components constant, the effect of the chitosan concentration on the biosensor response was investigated. The results are presented in Figure S3 and Table S1. Accordingly, the most sensitive (0.6398 μA/mM) and precise (%CV = 2.02) biosensor responses were obtained with electrodes using a 0.1% chitosan solution. It can be observed that the biosensor response increased when the chitosan concentration was increased from 0.05 to 0.1% and then gradually decreased. In fact, electrodes prepared with a 1% chitosan solution suffered from structural disruption shortly after preparation, where the bioactive layer detached from the electrode entirely. The decrease in the biosensor response with increasing chitosan concentration could be attributed to the densely porous structure of chitosan potentially hindering the enzyme–substrate interaction. Looking at the literature data, it is worth noting that chitosan is often used within the concentration range of 0.05 to 0.2%.^34−38^ In this study, with the developed biosensor, maximum responses were obtained within the same range and the optimal chitosan quantity was selected as 0.1%.

#### 3.3.3 Effect of Nafion Amount on Biosensor Response

The optimization of the Nafion concentration has been carried out, and the optimal Nafion amount was found to be 0.1%. Upon examination of Figure S4 and Table S2, it is evident that increasing the Nafion concentration from 0.05 to 0.1% results in an enhancement of biosensor response, followed by a gradual decrease. In this regard, it has yielded outcomes similar to chitosan. Although Nafion is a conductive polymer, increasing its concentration has led to denser and more compact layers. This situation might have formed a barrier to the substrate–enzyme interaction.

#### 3.3.4 Optimization of Drop Volume

To investigate the effect of the immobilization components’ drop volume on the biosensor response and determine the optimal drop volume, volumes of 2.5, 5, 7.5, and 10 μL were tested on the electrode surface. According to the results shown in Table S3 and Figure S5, when the drop volume increased from 2.5 to 5 μL, the biosensor response also increased. However, for volumes >5 μL, there was no significant change in biosensor sensitivity. The surface of the working electrode was not fully covered with a 2.5 μL drop volume, while it was fully covered with a 5 μL volume. For 7.5 and 10 μL volumes, a portion of the liquid overflowed from the surface of the working electrode. The obtained responses indicate that a 5 μL drop volume is sufficient for the working electrode, and an excess did not increase the biosensor sensitivity since it exceeded the surface area of the working electrode. In the experiments conducted with the same electrode, no optimization study was conducted for the droplet volume. Heo et al.^139^ 7 μL, López-Marzo and Baldrich^39^ 10 μL, Zhybak et al.^40^ 2 μL, El Harrad and Amine^31^ used 5 μL drop volume.

#### 3.3.5 Effect of GNP on Biosensor Response

**Figure 7:**
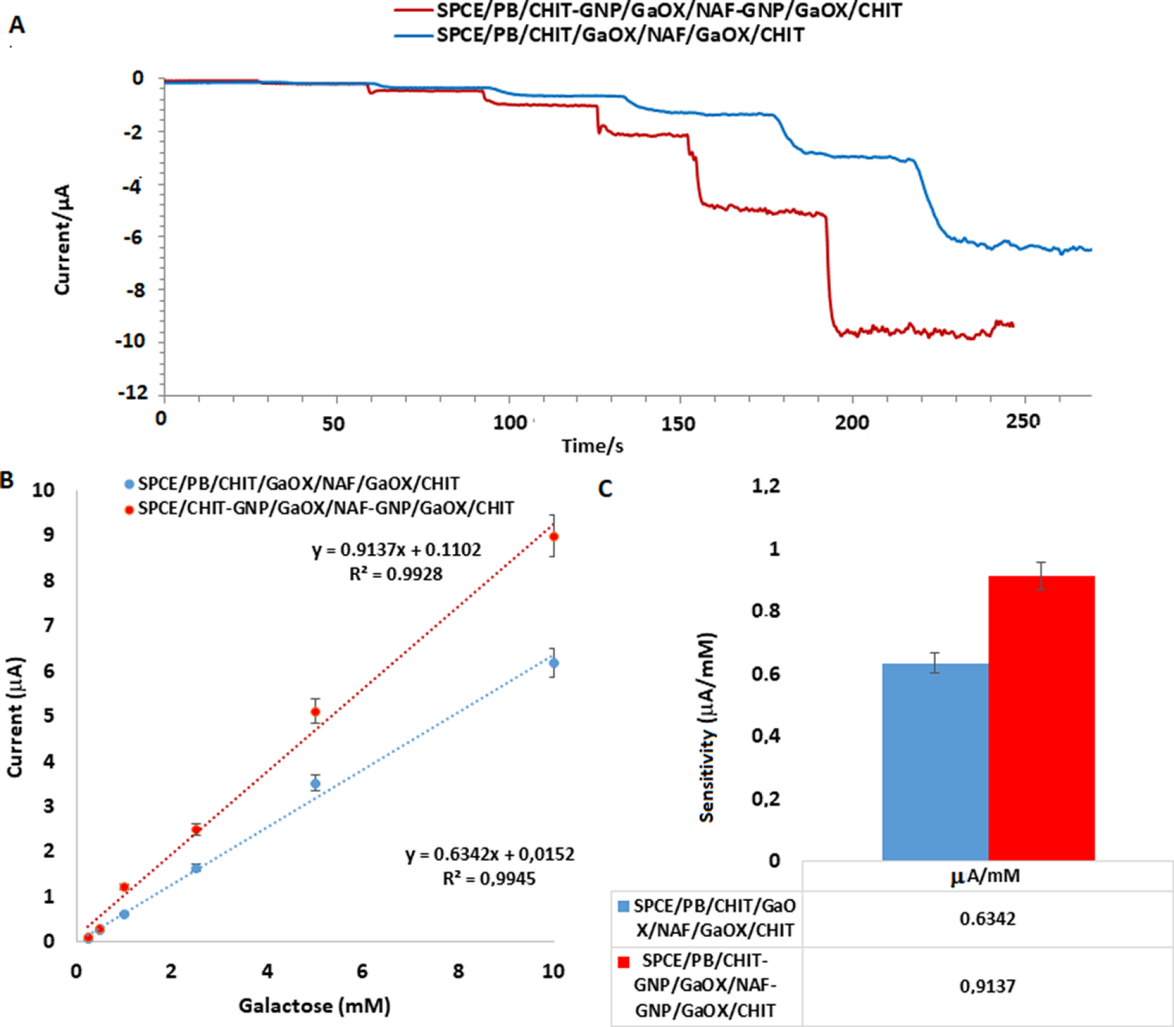
Effect of GNP on the biosensor response. Amperometric results (A) for the addition of 0.2–10 mM galactose to SPCE/PB/CHIT-GNP/GaOX/NAF-GNP/GaOX/CHIT and SPCE/PB/CHIT-GNP/GaOX/NAF-GNP/GaOX/CHIT electrodes, calibration graphs (B) corresponding to these results, and a column chart (C) comparing their sensitivities.

Recent studies, particularly those conducted in the last 20 years, have demonstrated that GNPs have garnered significant interest in the field of electrochemical sensors, as well as in many other areas, owing to their unique electronic, optical, and catalytic properties. The combination of GNP’s conductivity and an exceptionally high surface area-to-volume ratio has enabled excellent sensitivity and selectivity in measurements, as it promotes strong and abundant binding of adsorbed species. Indeed, Figure 7A shows that the incorporation of GNP into the immobilization method results in a decrease in the number of redox peaks due to the increased enzyme binding on the surface. In Figure 7, a comparison between GNP-modified and nonmodified electrodes is shown, where the surface area of the biosensor has been calculated, demonstrating a 26% increase in surface area attributed to GNP. The impact of GNP on the biosensor response is illustrated in Figure 7B,C. The addition of GNP has enhanced the biosensor response by approximately 1.5 times, which is an expected outcome given the properties of GNP.

#### 3.3.6 Optimization of Galactose Oxidase Concentration

Results regarding the amount of GaOX are presented in Table S4 and Figure S6. Typically, in enzyme-based biosensors, as the amount of enzyme increases, the sensitivity of the biosensor increases up to a certain point. Beyond that, due to the diffusion barrier created on the electrode surface, the biosensor response remains constant or decreases.^41,42^ The obtained results also demonstrate this phenomenon. Upon examination of the results, it can be observed that initially, as the enzyme amount increases, the biosensor response dramatically increases. In biosensors employing 10 and 20 mg/mL GaOX, the most sensitive biosensor responses are achieved, and there is no significant difference between the responses obtained from these two concentrations. The activity of GaOX used in this study is 3 U/mg, while it is 0.3 U on the electrode surface.

### 3.4 Working Temperature and pH Effect on Biosensor Responses

Upon examining Figure S7A, it will be seen that the developed free galactose biosensor does not exhibit activity below pH 5, starts to increase from pH 6, reaches a maximum at pH 7.5, and shows 95% activity at pH 8. The source of GaOX used in this study is *Dactylium dendroides (D. dendroides)*. These obtained results are in line with the literature.^43^. This also demonstrates the immobilization method does not alter the enzyme’s optimum pH. As shown in Figure S7B, when the temperature increases from 15 to 25 °C, the biosensor response also increases dramatically and reaches a peak. However, after 35 °C, the biosensor response gradually decreases. Paukner and colleagues found the optimum temperature for GaOX isolated from *D. dendroides* to be 35 °C. At 30 °C, the enzyme’s activity remains stable for 24 h, while at 40 °C, it halves in 11.2 min, and at 60 °C, it halves in 2.7 min.^43^ In this study, it has been found that the immobilization method developed slightly reduces the optimum temperature of GaOX. It is expected that immobilized enzymes would have slightly different optimum temperatures and pH values compared with free enzymes. Due to the lower process stability of the enzyme at higher temperatures, the optimum temperature has been determined as 25 °C. Thus, with the developed biosensor, measurements can be conducted at room temperature, making the use of the biosensor quite practical.

### 3.5 Analytical Characteristics of the Biosensor

#### 3.5.1 Linear Range

**Figure 8:**
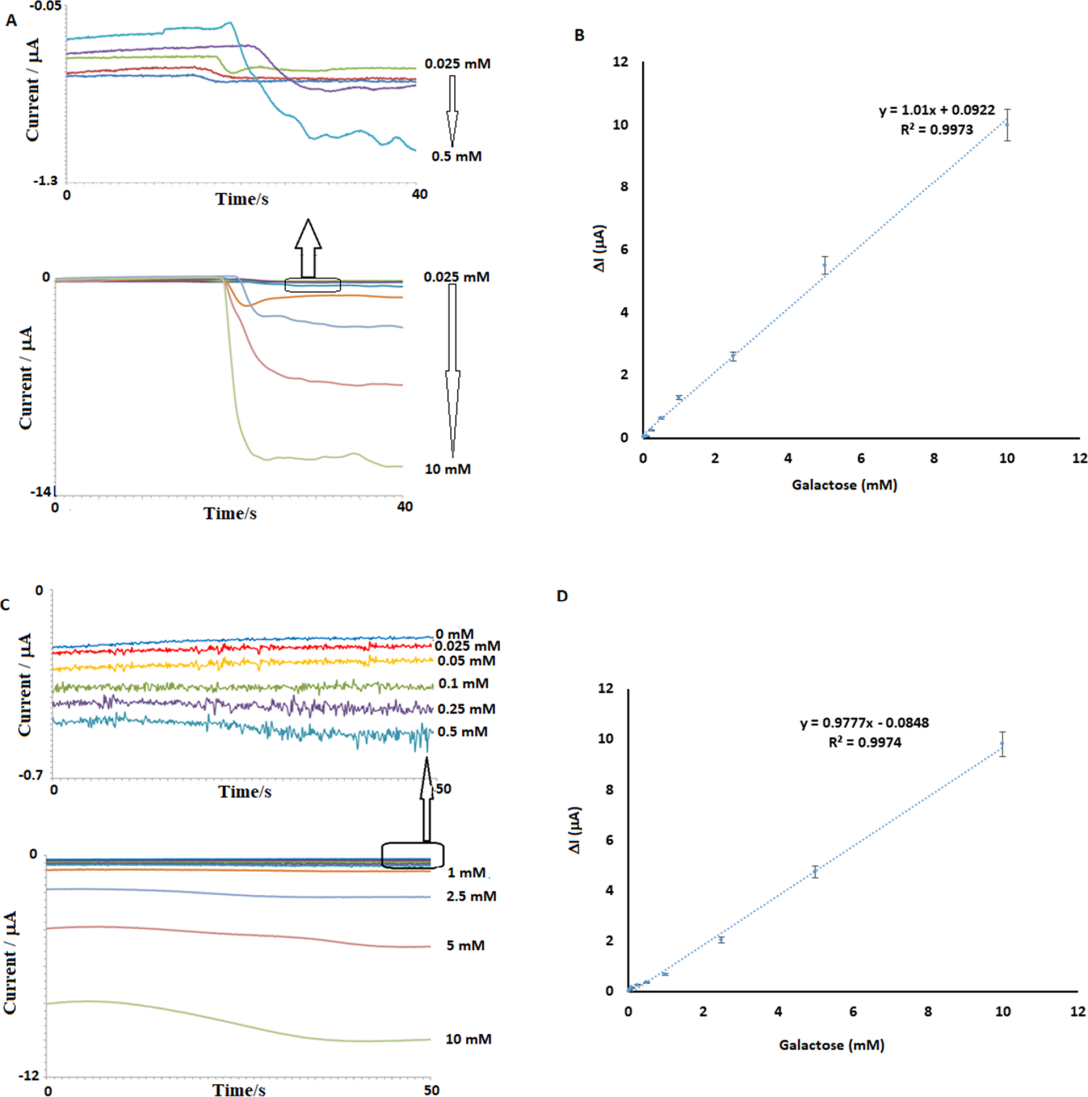
Amperometric results for determining the linear detection range of the biosensor. Amperometric responses using the stirred method for galactose concentrations ranging from 0.025 to 10 mM (A) and a related current-concentration linear fitting curve (B). Amperometric responses using the drop-cast method for galactose concentrations ranging from 0 to 10 mM (C) and related current-concentration linear fitting curve (D).

It is important for the developed biosensor to measure plasma/serum galactose levels. The linear detection range of the developed free galactose biosensor was determined to be 0.025–10 mM (Figure 8). According to literature data, plasma galactose levels are considered normal below 0.264 mM in newborns, and below 0.11 mM for infants over 15 days old and adults.^44^ In the stirred method (Figure 8A), the sample is diluted 10 times, while in the drop-cast method (Figure 8B), it is diluted 2 times. This condition requires a minimum plasma concentration of 0.25 mM for quantification in the mixing method and 0.05 mM for the drop-cast method. Therefore, for the inclusion of healthy sample measurements, it is more accurate to use the drop-cast method. Plasma galactose levels in galactosemia patients can exceed 0.55 mM and reach levels of up to 6 mM. From this perspective, the determined range of the developed free galactose biosensor covers the plasma galactose levels of patients as well as a significant portion of healthy individuals. For the standard curve plotted in the stirred method, the equation is *y* = 1.01*x* + 0.0922 with an *R*^2^ value of 0.9973 (Figure 8A), while for the dropwise method, the equation is *y* = 0.977*x* – 0.0848 with an *R*^2^ value of 0.9974 (Figure 8B).

#### 3.5.2 Repeatability and Reproducibility

A repeatability test was performed both intraday and interday by conducting six replicates at three points of low (0.1 mM), medium (1 mM), and high (10 mM) galactose concentrations. The average value (*x*), standard deviation (SD ±), and %CV values are provided in Table 1. The obtained results for both intraday and interday reproducibility are below 15%, which is deemed acceptable.

**Table 1:** Findings on Intraday and Interday Repeatability of the Developed Biosensor

concentration (mM)	intraday repeatability (*n* = 6)		interday repeatability (*n* = 6)	
	mean ± SD	CV%	mean ± SD	CV%
0.1	0.096 ± 0.0054	5.63	0.104 ± 0,0108	10.38
1	1.041 ± 0.047	4.51	1.024 ± 0.098	9.57
10	10.025 ± 0.34	3.39	9.946 ± 0.74	7.44

For reproducibility, biosensors prepared with six different electrodes were studied and the biosensor responses to galactose concentrations between 0.025 and 10 mM were graphed. The %CV value calculated for the reproducibility of the graphs from the slopes was found to be 7.78%.

#### 3.5.3 Substrate Specifity and Interference Effects

**Figure 9:**
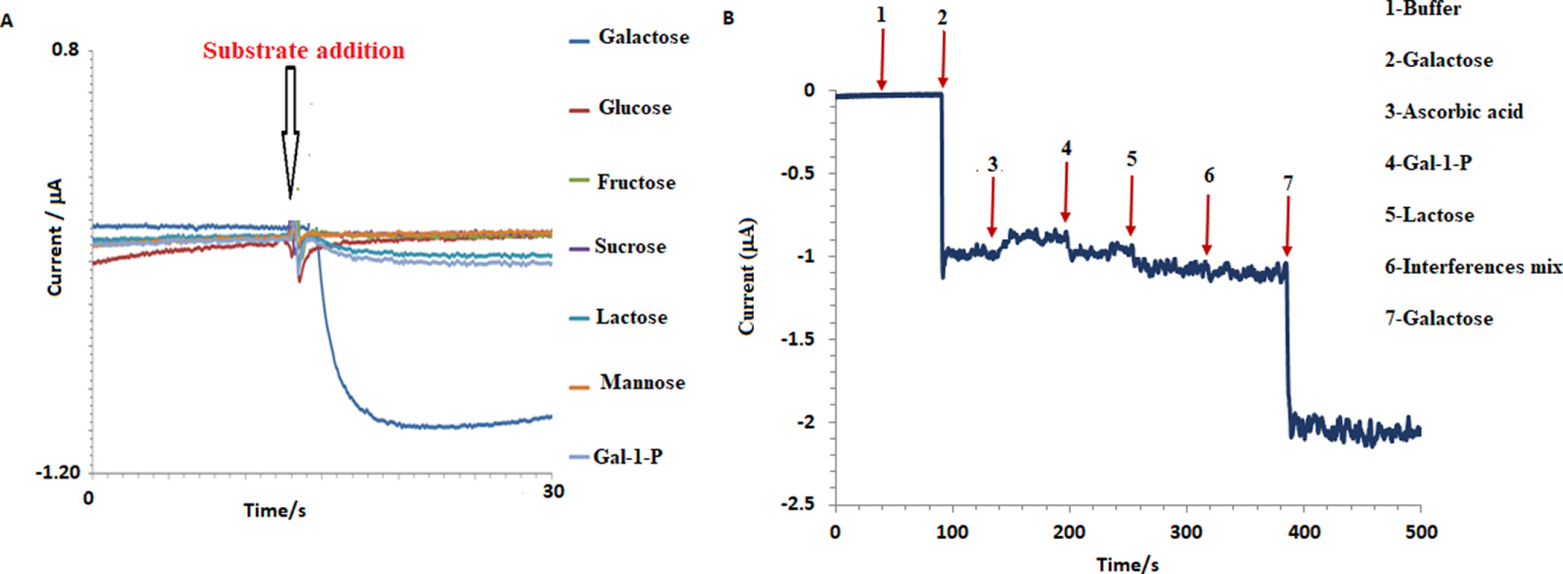
Amperometric results regarding the substrate specificity of the biosensor. The concentration of each substrate is 1 mM (A). Amperometric responses were determined for the interference test of the developed free galactose biosensor. Galactose: 1 mM; lactose: 1 mM; gal-1-P: 1 mM; ascorbic acid (A.A) 200 μM; interferences mix: 5 mM KCl, 1 mM MgCl_2_, 5 mM urea, 10 mM uric acid, 5 mM glucose, 5 mM creatinine, 100 mM creatine, 150 mM NaCl, 5 mM CaCl_2_, 250 μM lactate, 250 μM pyruvate, 250 μM FeCl_3_, 1 mM ATP, 400 μM cysteine, 200 μM Cu(CH_3_COO)_2_, and 10 μM glutathione (GSH) (B).

For the purpose of determining the substrate specificity of the developed free galactose biosensor, other sugars and sugar derivatives structurally similar to galactose, including glucose, fructose, mannose, sucrose, lactose, and galactose-1-phosphate, were tested. Each analyte, including galactose, was concentrated to 1 mM in solution. Figure 9A shows the amperometric responses for this experiment, while Table 2 displays a comparison of biosensor response to the substrate. The response to galactose was considered as 100%, and the responses to other analytes were calculated as a percentage of the response to galactose, determining the % biosensor response. As a result, the developed biosensor did not respond to glucose, fructose, mannose, and sucrose, which do not contain galactose in their structure. However, for lactose, a disaccharide containing galactose, and galactose-1-phosphate, a phosphorylated sugar, the biosensor responded with approximately 10% of the response to galactose.

**Table 2:** Substrate Selectivity and Interference Effect of Some Compounds on Biosensor Response

substrate	biosensor response %
galactose	100
glucose	0
fructose	0
sucrose	0
lactose	9.75 ± 0.42
mannose	0
gal-1-P	11.95 ± 0.48

In interference studies, it is desirable for the concentrations of the species to be tested to be above the average values.^45^ The following species did not produce a measurable response in both the galactose-free buffer solution and the 1 mM galactose-containing buffer solution: 5 mM KCl, 1 mM MgCl_2_, 5 mM urea, 10 mM uric acid, 5 mM glucose, 5 mM creatinine, 100 mM creatine, 150 mM NaCl, 5 mM CaCl_2_, 250 μM lactate, 250 μM pyruvate, 250 μM FeCl_3_, 1 mM ATP, 400 μM cysteine, 200 μM Cu(CH_3_COO)_2,_ and 10 μM glutathione (GSH) (Figure 9B). In Figure 9B, it can be observed that the biosensor responds slightly to ascorbic acid, in addition to its natural substrates galactose, lactose, and galactose-1-phosphate. In this study, the species present in the interferent mixture were tested only in the presence of galactose, and none of them exhibited any interference with the measurement. Only, the substances that had an impact on the biosensor response were gal-1-P, lactose, and ascorbic acid. Among these, gal-1-P and lactose caused an approximately 10% increase, while the presence of ascorbic acid caused a decrease of approximately 11% in the biosensor response.

#### 3.5.4 Sample Analysis and Recovery

The recovery test of the developed free galactose biosensor was performed by spiking the healthy control plasma pool with standard additions for three levels. According to the results shown in Table 3, the recovery rate ranges from 93 to 100%.

**Table 3:** Findings of the Recovery Test for the Free Galactose Biosensor

added galactose (mM)	found galactose (mM)	recovery%	VK%
0	0.031 ± 0.00186		
0.1	0.124 ± 0.0062	93.00	5
1	0.97 ± 0.0291	93.90	3
10	10.059 ± 0.402	100.28	3.99

The developed free galactose biosensor was tested for practical use by working with 10 healthy and 11 patient samples that reflect plasma galactose levels. The drop-cast method was used for both healthy and patient samples. In the stirred method, the sample is diluted by a factor of 10 in the working buffer. This level is below the galactose concentration that the biosensor can measure for some healthy individuals. Therefore, the sample application trial was conducted using the drop-cast method, and the amperometric responses related to this are presented in Figure S8A. The results and method comparison based on these responses are provided in Figure S8B.

#### 3.5.5 Stability and Comparison of the Galactose Biosensor

In this test conducted to assess the storage stability of the developed free galactose biosensor, measurements were taken for 1 mM galactose. Two electrodes were prepared, one of which was stored at +4 °C when not in use and the other at room temperature. According to the results, when the biosensor was stored at +4 °C, a 50% loss of activity was observed after 12 weeks, while when stored at room temperature, a 50% loss of activity was observed after 7 weeks.

Table 4 includes the performance characteristics of existing biosensors in the literature targeting galactose measurement in serum/plasma/blood. According to this, the developed free galactose biosensor in this study has an average determination range, a fast response time, and quite good storage stability.

**Table 4:** Comparison of This Study with the Galactose Biosensors Available in the Literature^a^

electrode	sensitivity	linear range (mM)	response time (s)	stability (day)	sample	references
GaOx/CA/Co-SPCE	7.0 μA mM^–1^ cm^–2^	0.1–25.0	30	14	serum	(46)
GalOx/glu/PC/H_2_O_2_		0.0–28.0	40	7	plasma, blood	(47)
Glu/GalOx/glu/1,3-DAB/res/Pt		50 × 10^–3^–6.0	18	30	plasma	(48)
GaOx/Glu/CHIT/PB/Pt	49.0 nA mM^–1^	0.1–6.0	42–60	30	serum	(49)
GaOx/Co_3_O_4_/graphene/GCE	6.6 μA mM^–1^ cm^–2^	9.0 × 10^–3^–0.6	15	∼30	serum	(50)
GaOx/Co_3_O_4_/MWCNTs/GCE	10.4 μA mM^–1^ cm^–2^	9.0 × 10^–3^–1.0	20	∼30	serum	(50)
[50]GaOx/collagen/H_2_O_2_	1.0–3.0 mA M^–1^	5.0 × 10^–4^–0.6	60	∼300	serum	(51)
GaOx/glu/CHIT/SWCNT-GCE	1126.0 nA Mm^1–^	up to 1.0			blood	(52)
GaOx/polypyrrole/Pt	3.5–14.7 mA M^–1^ cm^–2^	5.0 × 10^–4^–2.0		15	blood	(53)
GaOx/polypyrrole-[p(HEMA)]/Pt	937.0 μA M^–1^	5.0 × 10^–2^–10.0	70	270	serum	(54)
Nafion/PU/xerogel-GaOX/Pt	0.037 μA mM^–1^	0.5–7.0	∼30		serum	(15)
**CHIT/GaOX/NAF/PB/SPCE**	**1.01** μA mM^–1^	**0.025–10**	**20**	**84**	**plasma**	**this study**

a The values written in bold are the values of this study.

## 4 Conclusions

The operational principle of the developed free galactose biosensor is based on the reduction of H_2_O_2_, an electroactive compound generated as a result of galactose oxidase activity, through electrodeposition of PB onto the SPCE at a low potential. The low potential reduction of H_2_O_2_ reduces the interference effects of electroactive species, such as ascorbic acid and uric acid. The immobilization method applied in this study is unique. The immobilization method relies on attaching the enzyme structure to the electrode surface through electrostatic forces between chitosan and Nafion layers. The developed biosensor exhibits minimal interference, accurate measurement capabilities, repeatability, fast response time, and ease of preparation. In this study, the biosensor was designed for multiple uses, not just single-use. If a disposable design were desired, covalent immobilization techniques would have been attempted. In that case, there would be no membrane between the enzyme and the substrate, leading to more sensitive biosensor responses. However, biosensors using covalent immobilization techniques tend to have very short storage stability. The developed biosensor was able to measure target analytes in the target matrices, thus achieving the main goal of this study.

## Supporting Information Available

Cyclic voltammograms of the electrodeposition of the PB; amperometry responses related to concentration range working in optimization studies; findings showing the optimization of chitosan concentration; findings showing the optimization of Nafion concentration; findings showing the optimization of the dropping volume; findings showing the optimization of GaOX concentration; findings showing optimization of pH and temperature; and amperometric responses of healthy controls and patients with galactosemia obtained by drop method (PDF)
