# Supplementary material for: Development of a New Amperometric Biosensor for Measurement of Plasma Galactose Levels
Source: ACS Omega. 2024 Feb 7;9(7):7621–33. doi: 10.1021/acsomega.3c06789 (PMC10882682; doi:10.1021/acsomega.3c06789)
Supplement: Supplementary file 1 — ao3c06789_si_001.pdf [file ao3c06789_si_001.pdf]

SUPPLEMENTARY MATERIALS

DEVELOPMENT OF A NEW AMPEROMETRIC BIOSENSOR FOR MEASUREMENT OF PLASMA GALACTOSE LEVELS

Erhan CANBAY [1*], Ebru SEZER [1], Ebru CANDA [2], Havva Yazıcı [2], Sema KALKAN UÇAR[2], Mahmut ÇOKER [2], Eser YILDIRIM SÖZMEN [1]

[1] Department of Medical Biochemistry, Faculty of Medicine, Ege University, Bornova- Izmir, 35100, Turkey

[2] Department of Pediatric Metabolic Disease, Faculty of Medicine, Ege University, Bornova, Izmir, Turkiye

Keywords: Galactose, Biosensor, Galactose oxidase, Amperometric, galactosemia

***Corresponding Author:** Ph.D. Erhan CANBAY

Ege University, Faculty of Medicine

Department of Medical Biochemistry

35100 Bornova-İzmir/ TURKEY

E-mail: erhancanbay87@gmail.com ;

biochem.erhancanbay@gmail.com ;

erhan.canbay@ege.edu.tr ;

Tel: +90 232 390 36 99

Table of Contents

FIGURES

Figure S1. Cyclic voltammograms of the electrodeposition of the PB taken between -0.2V & 1.0V……………………………………………………………………………………...**3**

Figure S2. Amperometry responses related to concentration range working in optimization studies…………………………………………………………………………..…..**4**

Figure S3. . The graph compares the sensitivities of modified SPCEs with different amounts of chitosan………………………………………………………………………………...…**5**

Figure S4. The graph compares the sensitivities of SPCEs modified with different amounts of nafion………………………………………………………………………………….…**6**

Figure S5. Comparison of the sensitivities of modified SPCEs with different drop volumes..**7**

Figure S6. Comparison of the sensitivities of modified SPCEs with different GaOX concentrations………………………………………………………………………………**8**

Figure S7. Results for pH and temperature optimization of the biosensor……………………**9**

Figure S8. The amperometric responses of healthy controls and patients with galactosemia obtained by drop method……………………………………………………………...…**10**

TABLES

Table S1. Data on the effect of chitosan amount on biosensor response……………………..**5**

Table S2. Data on the effect of nafion amount on biosensor response………………………**6**

Table S3. Findings on optimization of drop volume…………………………………………**7**

Table S4. Findings on GaOX amount optimization…………………………………………..**8**

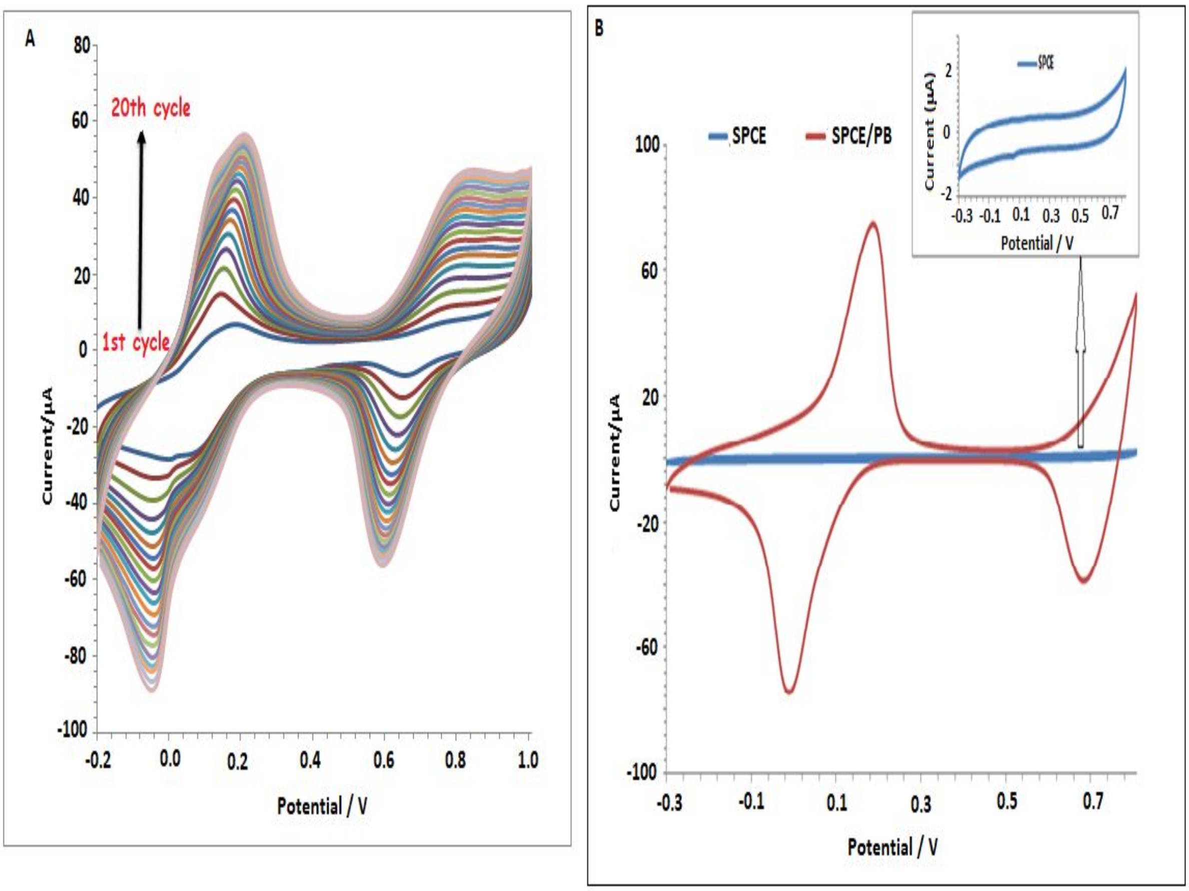

Fig. S1. Cyclic voltammograms of the electrodeposition of the PB taken between -0.2V & 1.0V (scan rate 50 mV/s). Measurements were taken in a mixture containing 2.0 mM $FeCl_3$, 2.0 mM $K_3Fe(CN)_6$, 0.1 M KCl and 20 mM HCl (A). Cyclic voltammograms taken with SPCE and SPCE/PB electrodes in 50 mM pH:7.0 PBS containing 0.1 M KCl (B).

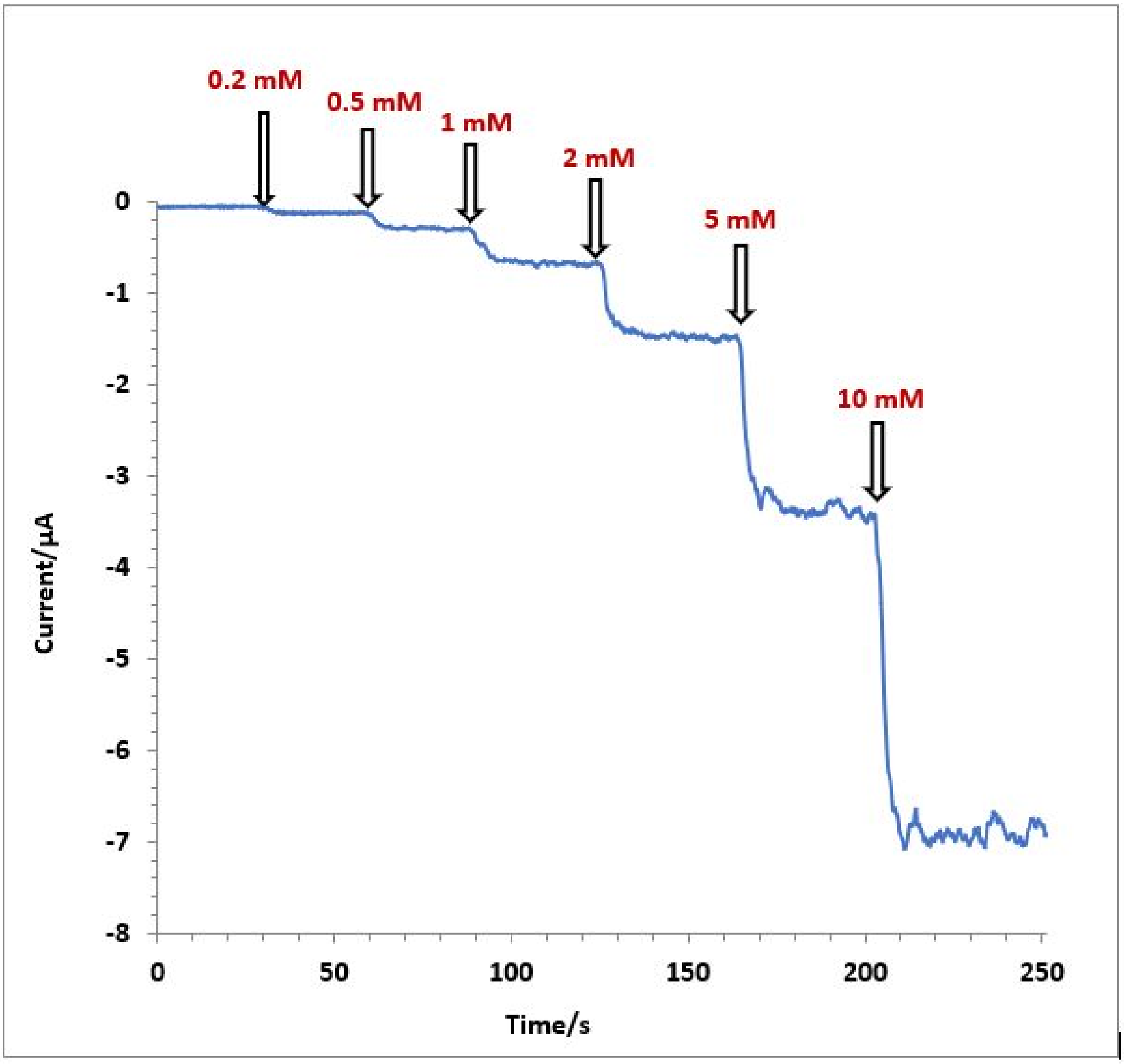

Fig. S2. Amperometry responses related to concentration range working in optimization studies. Amperometry results of the free galactose biosensor (SPCE/PB/CHIT /GaOX/NAF /GaOX/CHIT) for 0.2-10 mM galactose addition. Measurements were taken in 50 mM pH:7.5 PBS containing 0.1 M KCl. Amperometric measurements taken at -0.05 V.

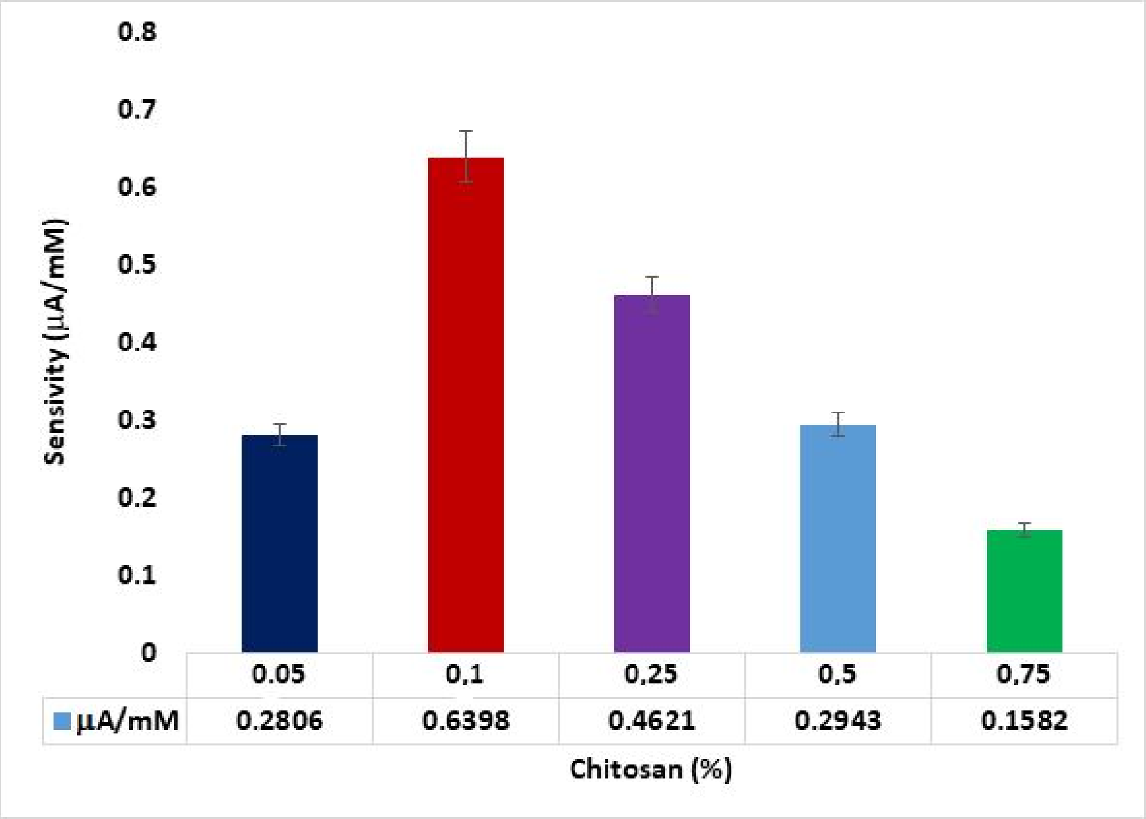

Fig. S3. The graph compares the sensitivities of modified SPCEs with different amounts of chitosan for determination of galactose concentration in the 0.2-10 mM concentration range..

Table S1. Data on the effect of chitosan amount on biosensor response.

Chitosan (%)	Equation	R^2	CV%
0.05	y=0.2806x + 0.087	0.991	2.54
0.1	y=0.6398x + 0.322	0.992	2.02
0.25	y=0.4621x+0.233	0.990	2.47
0.5	y=0.2943x – 0.0851	0.981	5.62
0.75	y=0.1582x – 0.0216	0.970	12.54

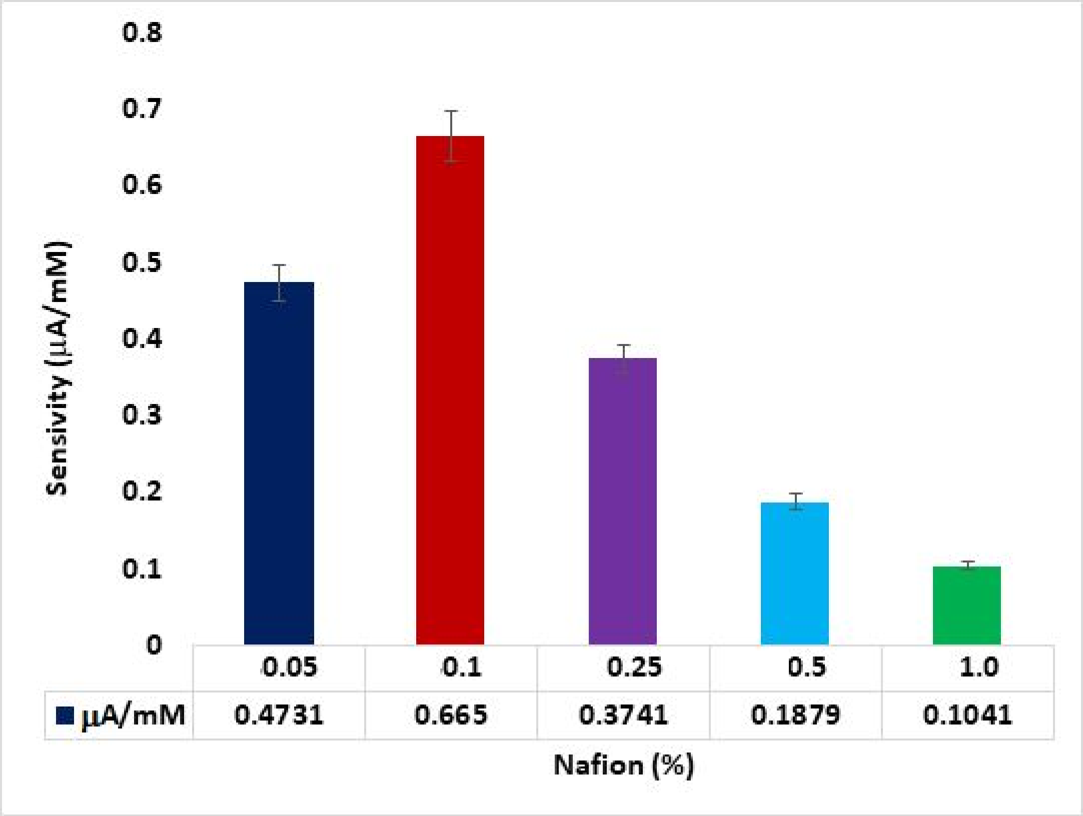

Fig. S4. The graph compares the sensitivities of SPCEs modified with different amounts of nafion for determination of galactose concentration in the 0.2-10 mM concentration range..

Table S2. Data on the effect of nafion amount on biosensor response

Nafion (%)	Equation	R^2	CV%
0.05	y=0.4731x + 0.1426	0.995	3.05
0.1	y=0.6665x – 0.0865	0.998	2.74
0.25	y=0.3741x+0,4218	0.995	3.51
0.5	y=0.1879x + 0.1015	0.9794	9.58
0.75	y=0.1041x + 0.2829	0.9303	13.52

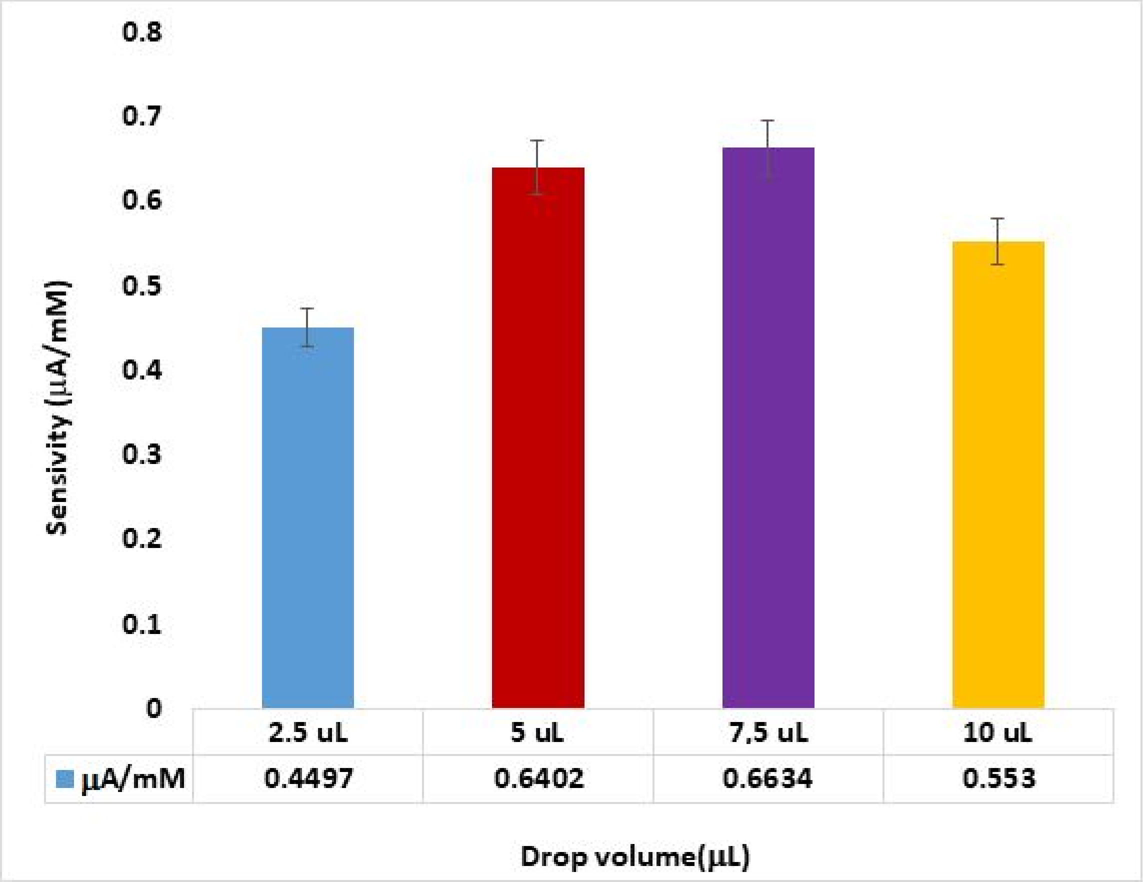

Fig. S5. Comparison of the sensitivities of modified SPCEs with different drop volumes for the determination of galactose concentration in the 0.2-10 mM concentration range

Table S3. Findings on optimization of drop volume

**Drop volume (µL)**	**Equation**	**R^2**	**CV%**
2.5	y=0.4497x + 0.7131	0.983	6.08
5	y=0.6402x + 0.7948	0.990	4.74
7.5	y=0.6634x+0.6715	0.986	4.52
10	y=0.5533x + 0.7057	0.985	5.42

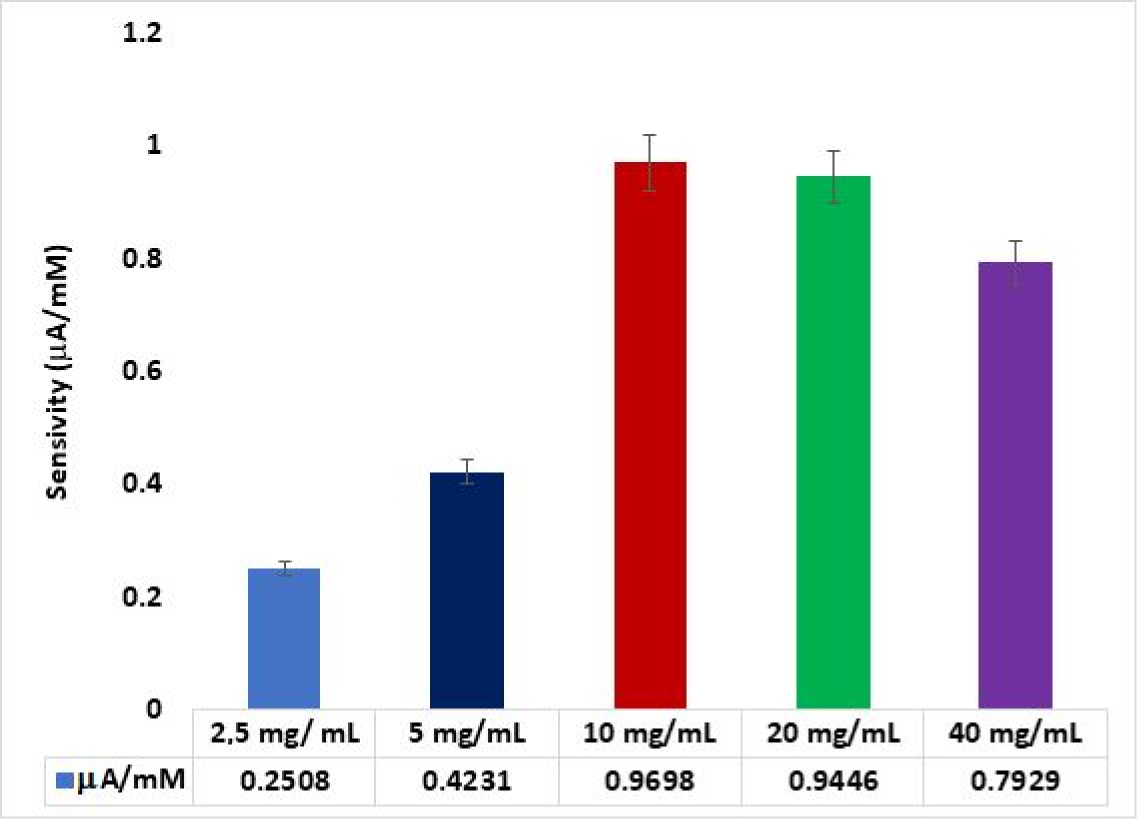

Fig. S6. Comparison of the sensitivities of modified SPCEs with different GaOX concentrations for the determination of galactose concentration in the 0.2-10 mM concentration range

Table S4. Findings on GaOX amount optimization

GaOX concentration (mg/mL)	equation	R^2	CV%
2.5	y=0.2508x – 0.0446	0.982	7.47
5	y=0.4231x + 0.2401	0.992	4.33
10	y=0.9698x + 0.0145	0.995	2.71
20	y=0.9446x + 0.1062	0.997	1.97
40	y=0.7929x – 0.1052	0.983	6.54

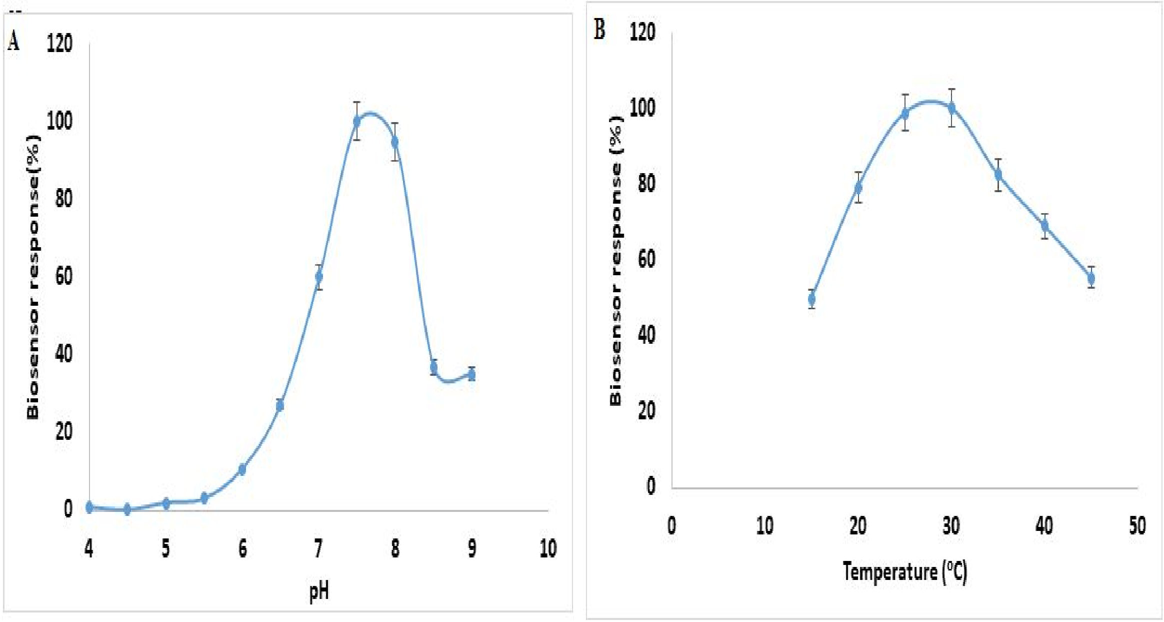

Figure S7. Results for pH optimization of the biosensor (A) Results for the temperature optimization of the biosensor (B). 3 replicate measurements were taken for the 1 mM galactose concentration

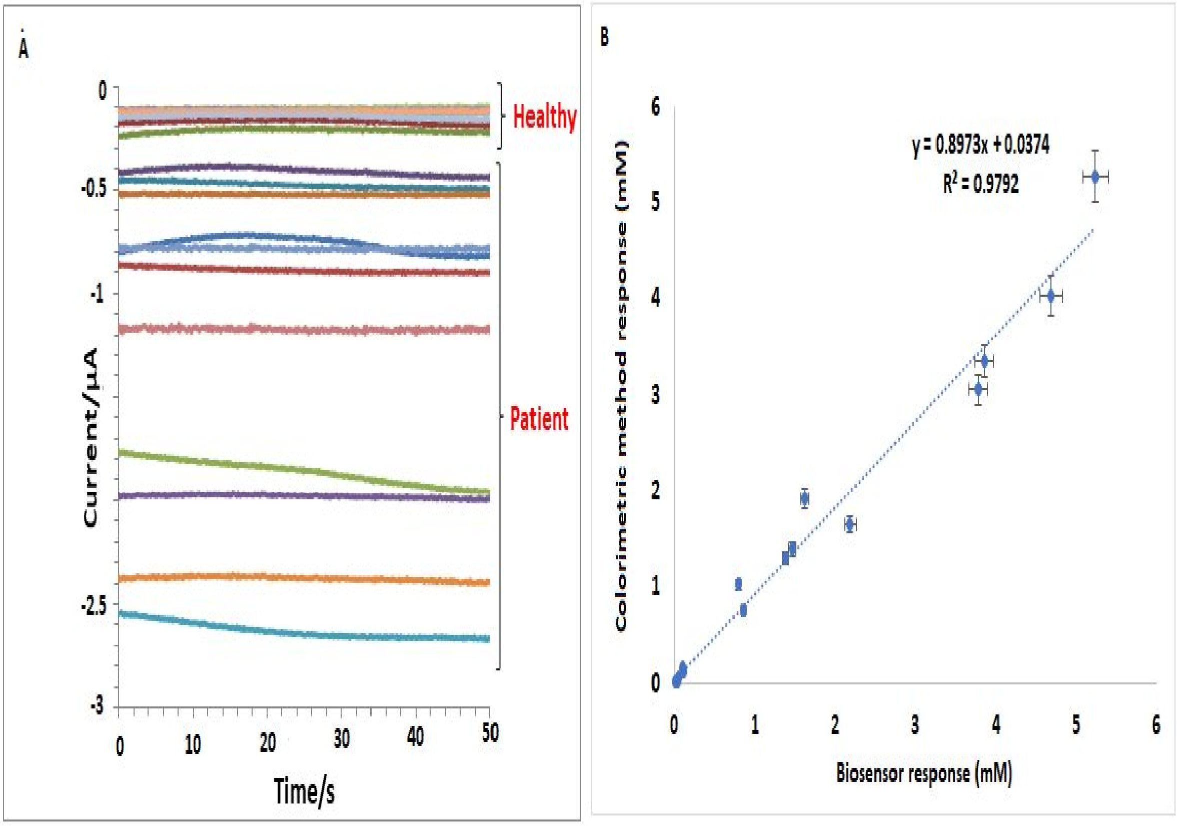

Figure S8. The amperometric responses of healthy controls and patients with galactosemia obtained by drop method (A). Correlation graph drawn for method comparison (B)
